# Usefulness and growing need for intraoperative transthoracic echocardiography: a case series

**DOI:** 10.1186/s12871-015-0066-0

**Published:** 2015-06-10

**Authors:** Kotaro Hori, Tadashi Matsuura, Takashi Mori, Kiyonobu Nishikawa

**Affiliations:** Department of Anesthesiology, Osaka City University Graduate School of Medicine, 1-5-7 Asahimachi, Abenoku, Osaka City, Osaka 545-8586 Japan

**Keywords:** Intraoperative monitoring, Transthoracic echocardiography, Transesophageal echocardiography

## Abstract

**Background:**

Physician-performed transthoracic echocardiography (TTE) is still seldom used during anesthesia. Despite its various advantages, there are only a few reports of intraoperative TTE. We report 3 cases in which intraoperative TTE was successfully used.

**Case presentation:**

A 75-year-old woman (Case 1) was scheduled for a posterior spinal fusion. When the wound was being closed, systolic blood pressure suddenly dropped to 30 mmHg. TTE revealed hypokinesis in the antero-septal region. Emergent coronary angiography showed 90 % stenosis in left anterior descending artery (Segment 7), and a bare metal stent was implanted. A 71-year-old woman (Case 2) with hypertrophic cardiomyopathy was scheduled for brain tumor operation. During anesthesia induction, the patient developed hemodynamic instability. TTE showed systolic anterior motion of the mitral valve, and appropriate treatment was administered. A 78-year-old woman (Case 3) was scheduled for revision total hip arthroplasty. When the wound was closed, TTE revealed severe hypovolemia despite massive infusion. We insisted on reopening the wound and found additional massive hemorrhage.

**Conclusion:**

Intraoperative TTE is a potent tool for quick hemodynamic evaluation because it is noninvasive and has sufficient diagnostic capabilities. The successful outcomes of our cases suggest the great usefulness of intraoperative TTE, and more frequent use is to be encouraged.

## Background

Physician-performed transthoracic echocardiography (TTE) during anesthesia has been used much less often than transesophageal echocardiography (TEE) despite TTE’s various advantages [1, 2]. It is probably because non-cardiologists are often inexperienced with TTE, and it is sometimes difficult to obtain adequate images with TTE during surgery, owing to mechanical ventilation and lack of access to some acoustic windows due to the need to maintain a sterile surgical field.

However, TTE use by non-cardiologists has been increasing, albeit mainly in emergency medicine [3] and intensive care units [4]. In anesthesiology, there are only a few reports regarding the utility of TTE, mainly in the preoperative period, and intraoperative use is rarely reported [5–7]. With respect to acoustic windows, recent studies have shown that diagnostic-quality TTE images can be obtained in almost all patients on mechanical ventilation [8, 9]. Furthermore, TTE can quickly evaluate hemodynamic state with focused views. One report noted that TTE permitted evaluation of the patient’s hemodynamic state within only 10 s [10]. TEE also has the potential to quickly evaluate the hemodynamics, but in some cases probe insertion into esophagus may be difficult, resulting in some loss of time.

The chief advantages of TEE are its high acoustic image quality and minimal invasiveness, which have led to its frequent intraoperative use. However, TEE is certainly minimally invasive, but it is not noninvasive. A recent study showed that the risk of TEE-associated major gastroesophageal injury was about 1 per 1000 patients, and the risk of mortality was about 1 per 5000 patients, suggesting reconsideration of the routine use of TEE [11]. In contrast, TTE is absolutely noninvasive and has no known adverse effects [12].

All of these findings suggest that intraoperative TTE could be more useful than is generally believed. Interest in intraoperative TTE is developing, but there are still a few reports of intraoperative TTE [1, 6, 7]. In this case series, we successfully used TTE for acute assessment of hemodynamics during anesthesia, leading to appropriate management. The successful outcomes of these cases suggest the usefulness of intraoperative TTE and support its more frequent use.

## Case presentation

### Case 1

A 75-year-old woman with an old compression fracture in the first lumbar vertebra was scheduled for a posterior spinal fusion. Her only known comorbidity was well-controlled hypertension and preoperative TTE was almost normal without asynergy. The operation was uneventful, but when the wound was being closed, her systolic blood pressure suddenly dropped to about 30 mmHg.

The surgeons quickly closed the wound and repositioned the patient to supine. Soon, we found active bleeding via the drainage tube, in which the hemorrhage volume was 1000 mL. We rapidly administered large amounts of fluids, but the patient’s blood pressure remained below 70 mmHg. Her hemoglobin level was 8.3 g/dl at the time. Because intraoperative electrocardiogram (ECG) monitoring showed ST elevation in lead II, we checked the 12-lead ECG, and evaluated the patient’s cardiac condition with TTE (Vivid S6; GE Healthcare; Amersham, Buckinghamshire, England). The ECG showed ST elevation in II, III, aVf, and V3–6. The TTE showed severe hypokinesis in the anteroseptal region at the mid-papillary level, and there was no apparent hypovolemia with sufficient left ventricular (LV) end-diastolic diameter. The cardiologist quickly concluded that emergency coronary angiography was necessary. The middle portion of left anterior descending artery (Segment 7) showed 90 % stenosis, and a bare-metal stent (MULTI-LINK VISION, 2.75 × 18 mm) was implanted.

### Case 2

A 71-year-old woman was scheduled for palliative surgical removal of a cerebellar metastasis from breast cancer to relieve her nausea and dizziness. She had a history of hypertrophic cardiomyopathy, which had been diagnosed 12 years previously and treated with atenolol. Atenolol was taken until the morning of surgery. On preoperative TTE, there was no apparent left ventricular outflow tract (LVOT) obstruction, and mitral regurgitation was trivial.

**Fig. 1:**
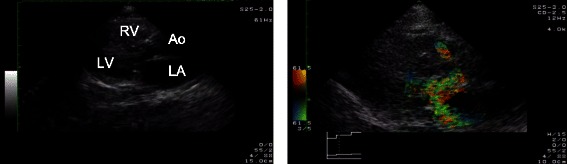
Intraoperative TTE view of Case 1 after tracheal intubation (parasternal long-axis view). A posteriorly directed jet of severe mitral regurgitation (a wall-hugging jet) and turbulent flow in the left ventricular outflow tract were seen with color Doppler (*right*). Further detailed evaluation of mitral valve motion was difficult because of acoustic window limitations (*left*). *LA* = left atrium, *LV* = left ventricle, *RV* = right ventricle, *Ao* = aorta

Anesthesia was carefully induced with low dose of thiopental, but the patient’s systolic blood pressure immediately dropped to 60–70 mmHg. No remarkable change was found in ECG monitor, including the heart rate. After tracheal intubation, TTE (Power vision 8000; Toshiba Medical Systems Corporation; Ohtawara, Tochigi, Japan) was performed while boluses of phenylephrine were repeatedly administered. We diagnosed systolic anterior motion (SAM) of the mitral valve on the basis of color Doppler findings and clinical course (Fig. 1). We quickly inserted a central venous catheter into the femoral vein for norepinephrine infusion to treat SAM. Soon after appropriate fluids and norepinephrine were administered, the patient’s blood pressure stabilized, and we decided to continue the surgery. We reduced the anesthetic dose after surgery, thereby decreasing the norepinephrine requirement. After extubation in the operating room, vasopressor infusion became unnecessary, and she required no circulatory support thereafter.

### Case 3

A 78-year-old woman with no apparent complications was undergoing revision total hip arthroplasty, and open reduction and internal fixation under general anesthesia. Because her intraoperative hemorrhaging was severe, large volumes of blood were transfused. The total hemorrhage at the time of wound closure was 2500 mL. Despite massive infusion of blood, hypotension was remained; her systolic blood pressure was approximately 60 mmHg. Considering the possibility of heart failure due to massive infusion, we performed TTE (Vivid S6), which revealed severe hypovolemia without cardiac dysfunction (very small LV end-diastolic diameter with good contraction). Although there was no apparent hemorrhage via drainage, we reported the TTE findings to the surgeons and insisted on reopening of the wound. This revealed approximately 1800 mL of additional venous hemorrhage. The surgeon achieved complete hemostasis and the patient did well thereafter.

## Discussion

The ability to quickly evaluate intraoperative hemodynamic conditions is an important skill for anesthesiologists. Intraoperative TTE is a potent tool for this purpose, using several focused views for acute assessment of hemodynamics [1, 2]. Furthermore, it is noninvasive and offers sufficient diagnostic capability. In this case series, we successfully evaluated various, unstable hemodynamic conditions with focused TTE views and were able to provide prompt and proper treatment for each patient.

Intraoperative TTE is one of the most useful monitoring methods for detecting myocardial ischemia, as in Case 1. Because myocardial ischemia is one of the main causes of perioperative mortality [13], intraoperative detection of it is very important. TTE has high sensitivity for myocardial ischemia and can even detect cases that are difficult to diagnose with ECG [14]. Furthermore, TTE can detect mechanical complication due to myocardial infarction, and these useful characteristics of TTE are also stated in the current guideline of acute coronary syndromes [15, 16]. Especially in the intraoperative period, it is often difficult to use 12-lead ECG because of surgical drape, TTE may provide more usefulness in the diagnosis for intraoperative myocardial ischemia.

The TTE findings in Case 1 suggested acute coronary syndrome and indicated subsequent percutaneous coronary intervention. In previous reports of intraoperative TTE [6, 7], hemodynamic management was the majority of changes as a result of TTE findings. Therefore, additional invasive procedures like percutaneous coronary intervention were rare outcome of TTE findings, which suggested the further need for intraoperative TTE.

Diagnosis of SAM of the mitral valve during anesthesia is also a typical scenario that suggests the usefulness of intraoperative TTE. Hypertrophic cardiomyopathy is conventionally classified as obstructive or non-obstructive, and many of these patients were non-obstructive at rest. However, it was recently reported that 70 % of hypertrophic cardiomyopathy patients developed obstruction with exercise or the Valsalva maneuver [17], which suggested that many hypertrophic cardiomyopathy patients could develop obstruction during anesthesia. Indeed, our patient developed SAM, even though her cardiomyopathy was diagnosed as non-obstructive in the preoperative TTE.

Hypertrophic cardiomyopathy obstruction is commonly diagnosed with echocardiography. Traditionally, SAM of the mitral valve is determined by M mode echocardiography, and measuring LVOT pressure gradient with continuous-wave Doppler is also common. However, in our case, these traditional methods were difficult to implement because of acoustic window limitation. Therefore, we indirectly diagnosed obstruction on the basis of color Doppler findings, an example of one scenario in which focused TTE views could be used for quick hemodynamic evaluation [6]. Because the cause of hypotension was identified by TTE, we could provide appropriate treatment and continue the surgery.

In Case 3, we were able to detect signs of residual active bleeding; consequently, an additional operation was performed to achieve complete hemostasis. As in Case 1, this case had a rare clinical course, in which an additional invasive procedure (additional operation in this case) was indicated by intraoperative TTE findings [6, 7]. Reliable and compelling findings are essential for the indication of an additional invasive procedure. Thus, this case illustrates the diagnostic usefulness of intraoperative TTE.

Of course, intraoperative TTE has some limitations. Although previous reports suggested that TTE was a valuable diagnostic tool whose use consumes little time [1–3, 10], using TTE often required some degree of training to obtain adequate images, especially for ventilated patients during general anesthesia. Furthermore, whether intraoperative TTE is preferable to TEE in a given case depends largely on surgical site or situation. For example, in the case of cervical surgery or regional anesthesia, TTE is preferable because TEE is difficult to use in such cases, but in the left lung surgery, TTE is not suitable. Additionally, when the images obtained by TTE are poor, TEE may be more useful which has high acoustic image quality. Intraoperative TTE is surely a useful tool for time-sensitive assessment, but other models of evaluation, including cardiology consultation and TEE, may sometimes be necessary.

## Conclusion

**Fig. 2:**
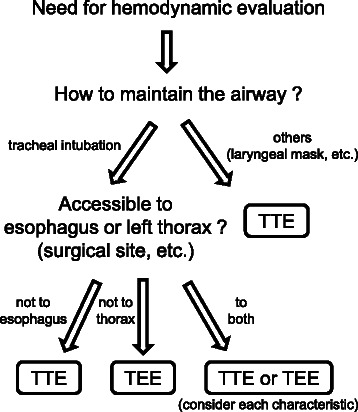
Algorithm for the choice of intraoperative echocardiography. First, if we maintain the airway other than tracheal intubation during surgery, we recommend *TTE* because it is difficult to quickly use *TEE*. Second, we can use *TTE* or *TEE* only if its acoustic window (patient’s left thorax or esophagus) are accessible. If both are available, we should choice more suitable one in a given case, considering each characteristic of *TTE* or *TEE*

We successfully used intraoperative TTE to rapidly provide diagnostic information, resulting in appropriate treatment decisions. Although intraoperative TEE is now frequently used with better image quality than TTE, we think that there are much more cases in which TTE use should be preferably considered because it is noninvasive and has sufficient diagnostic capabilities within a short time. The choice of TTE or TEE depends largely on the surgical site or situation, and therefore we finally suggested the algorithm for the choice of intraoperative echocardiography (Fig. 2). The less frequent use of intraoperative TTE may be mainly due to the lack of experience or training. Therefore, our suggested algorithm or a review of successful cases, such as those presented here, would be of considerable help for further use of intraoperative TTE.

## Consent

Written informed consents were obtained from the patients for publication of this Case report and any accompanying images. A copy of the written consent is available for review by the Editor of this journal.

## Abbreviations

TTE: Transthoracic echocardiography
TEE: Transesophageal echocardiography
ECG: Electrocardiogram
LV: Left ventricle
LVOT: Left ventricular outflow tract
LA: Left atrium
RV: Right ventricle
Ao: Aorta
SAM: Systolic anterior motion

## References

[1] Royse CF, Canty DJ, Faris J, Haji DL, Veltman M, Royse A (2012). Core review: physician-performed ultrasound: the time has come for routine use in acute care medicine. Anesth Analg.

[2] Cowie BS (2010). Focused transthoracic echocardiography in the perioperative period. Anaesth Intensive Care.

[3] Labovitz AJ, Noble VE, Bierig M, Goldstein SA, Jones R, Kort S, Porter TR, Spencer KT, Tayal VS, Wei K (2010). Focused cardiac ultrasound in the emergent setting: a consensus statement of the American Society of Echocardiography and American College of Emergency Physicians. J Am Soc Echocardiogr.

[4] Price S, Via G, Sloth E, Guarracino F, Breitkreutz R, Catena E, Talmor D (2008). Echocardiography practice, training and accreditation in the intensive care: document for the World Interactive Network Focused on Critical Ultrasound (WINFOCUS). Cardiovasc Ultrasound.

[5] Canty DJ, Royse CF, Kilpatrick D, Bowman L, Royse AG (2012). The impact of focused transthoracic echocardiography in the pre-operative clinic. Anaesthesia.

[6] Cowie B (2011). Three years' experience of focused cardiovascular ultrasound in the peri-operative period. Anaesthesia.

[7] Canty DJ, Royse CF (2009). Audit of anaesthetist-performed echocardiography on perioperative management decisions for non-cardiac surgery. Br J Anaesth.

[8] Jensen MB, Sloth E, Larsen KM, Schmidt MB (2004). Transthoracic echocardiography for cardiopulmonary monitoring in intensive care. Eur J Anaesthesiol.

[9] Vignon P, Chastagner C, François B, Martaillé JF, Normand S, Bonnivard M, Gastinne H (2003). Diagnostic ability of hand-held echocardiography in ventilated critically ill patients. Crit Care.

[10] Breitkreutz R, Walcher F, Seeger FH (2007). Focused echocardiographic evaluation in resuscitation management: concept of an advanced life support-conformed algorithm. Crit Care Med.

[11] Piercy M, McNicol L, Dinh DT, Story DA, Smith JA (2009). Major complications related to the use of transesophageal echocardiography in cardiac surgery. J Cardiothorac Vasc Anesth.

[12] Barnett SB, Kossoff G, Edwards MJ (1994). Is diagnostic ultrasound safe? Current international consensus on the thermal mechanism. Med J Aust.

[13] Slogoff S, Keats AS (1985). Does perioperative myocardial ischemia lead to postoperative myocardial infarction?. Anesthesiology.

[14] Autore C, Agati L, Piccininno M, Lino S, Musarò S (2000). Role of echocardiography in acute chest pain syndrome. Am J Cardiol.

[15] O’Gara PT, Kushner FG, Ascheim DD, Casey DE, Chung MK, de Lemos JA, Ettinger SM, Fang JC, Fesmire FM, Franklin BA, Granger CB, Krumholz HM, Linderbaum JA, Morrow DA, Newby LK, Ornato JP, Ou N, Radford MJ, Tamis-Holland JE, Tommaso CL, Tracy CM, Woo YJ, Zhao DX (2013). 2013 ACCF/AHA Guideline for the Management of ST-Elevation Myocardial Infarction: A Report of the American College of Cardiology Foundation/American Heart Association Task Force on Practice Guidelines. Circulation.

[16] Amsterdam EA, Wenger NK, Brindis RG, Casey DE, Ganiats TG, Holmes DR, Jaffe AS, Jneid H, Kelly RF, Kontos MC, Levine GN, Liebson PR, Mukherjee D, Peterson ED, Sabatine MS, Smalling RW, Zieman SJ (2014). 2014 AHA/ACC Guideline for the Management of Patients With Non–ST-Elevation Acute Coronary Syndromes: A Report of the American College of Cardiology/American Heart Association Task Force on Practice Guidelines. Circulation.

[17] Maron MS, Olivotto I, Zenovich AG, Link MS, Pandian NG, Kuvin JT, Nistri S, Cecchi F, Udelson JE, Maron BJ (2006). Hypertrophic cardiomyopathy is predominantly a disease of left ventricular outflow tract obstruction. Circulation.

